# How different sterols contribute to saponin tolerant plasma membranes in sea cucumbers

**DOI:** 10.1038/s41598-018-29223-x

**Published:** 2018-07-18

**Authors:** Emily J. S. Claereboudt, Igor Eeckhaut, Laurence Lins, Magali Deleu

**Affiliations:** 1 Biology of marine organisms and biomimetics, Research Institute for Biosciences, University of Mons, B-7000 Mons, Belgium; 2 Laboratory of molecular biophysics of interfaces, Gembloux Agro-Bio Tech, University of Liege, B-5030 Gembloux, Belgium

## Abstract

Sea cucumbers produce saponins as a chemical defense mechanism, however their cells can tolerate the cytotoxic nature of these chemicals. To elucidate the molecular mechanisms behind this tolerance a suite of complementary biophysical tools was used, firstly using liposomes for *in vitro* techniques then using *in silico* approaches for a molecular-level insight. The holothuroid saponin Frondoside A, caused significantly less permeabilization in liposomes containing a Δ^7^ holothuroid sterol than those containing cholesterol and resulted in endothermic interactions versus exothermic interactions with cholesterol containing liposomes. Lipid phases simulations revealed that Frondoside A has an agglomerating effect on cholesterol domains, however, induced small irregular Δ^7^ sterol clusters. Our results suggest that the structural peculiarities of holothuroid sterols provide sea cucumbers with a mechanism to mitigate the sterol-agglomerating effect of saponins, and therefore to protect their cells from the cytotoxicity of the saponins they produce.

## Introduction

Saponins form a large and diverse group of secondary metabolites produced by several terrestrial and marine organisms and are thought to be involved in chemical defense mechanisms. These natural products are well documented in plants^1^, but also occur in marine sponges^2,3^ and in two classes of echinoderms: Asteroidea or seastars^4,5^ and Holothuroidea or sea-cucumbers^6–9^. The term ‘saponin’ is derived from the Latin *sapo* (Engl.: soap) reflecting the amphiphilic detergent-like structure caused by the linkage of lipophilic isoprenoidal-derived aglycone to a hydrophilic saccharide moiety^10^.

Holothuroid saponins have been classified as being of the triterpenoid class^11^, and over 700 saponins have been described so far in Holothuroidea^12^. This large chemical diversity of saponins is paralleled with a wide variety of biological activities^13^ that includes but are not limited to hemolytic^14,15^, antibacterial^16^ antifungal^17,18^ cytotoxic^19,20^, ichthyotoxic^21^ and anti-tumoral^22^ activities.

Paradoxically, the biological roles of saponins in marine animals are still very speculative^13^ as are the molecular mechanisms behind these. Although saponins have been detected in most sea cucumber tissue, they seem to be particularly concentrated in the Cuverian tubules, a specialized defense system developed by some sea cucumber species belonging to the Holothuriidae family^23^. This localization of saponins is consistent with their cytotoxic effect on most organisms and their presumed chemical defense to repel predators^15^.

Most of these biological activities and roles result from the surface-active properties of saponins and the interactions they have with cellular membranes^24^. The ability of the glycosides to form complexes with 5,6-unsaturated sterols of target cell membranes is thought to determine their biological activity including ichthyotoxic action that may protect sea cucumbers against fish predation^21,25–27^. This complexing reaction leads to the formation of pores, permeabilization of cells and in the case of red blood cells the subsequent loss of haemoglobin in the extracellular medium^21,28^. Although the cytotoxic nature of saponins is well documented, the molecular mechanisms behind this activity is only beginning to be understood.

Animals that chemically defend themselves from predation must possess adaptations to circumvent auto toxicity (i.e., self-intoxication) which was very early attributed, in the case of sea cucumbers, to their fundamental difference in sterol composition^25^. Differences in liposomal permeability when in the presence of the holothuroid saponin Cucumaraoside G1 was observed for liposomes containing different sterols (including holothuroid sterols)^25^. The permeabilizing activity of the saponin decreased in the following order, in function of the sterol present: Cholesterol, total fraction of Δ5 sterol, total fraction of Δ7-sterols, Δ7-sterol xylosides and Δ5-sterol sulphates^25^. The collected data resulted in a proposed “sterol hypothesis” as the reason of tolerance of Holothuroidea to their own saponins^26^. It was hypothesized that perhaps the evolutionary replacement of Δ5-sterols, such as cholesterol with 5α-Cholest-7-en-3β-ol (Δ7) and 4α,14α-dimethyl-5α-cholest-9(11)-en-3β-ol (Δ9(11)), or other unusual sterols in sea cucumbers, could modulate the lytic action of the saponins they produce^25^. However, the molecular mechanisms explaining how this difference in sterol composition is able to protect sea cucumber cells, has never been investigated, let alone considered.

The aim of this study was therefore to elucidate the molecular mechanisms behind the tolerance of holothuroid to the cytotoxic saponins they produce. This investigation was conducted using complementary biophysical tools, using both *in silico* and *in vitro* approaches. As the complexity of living biological plasma membranes makes understanding the biophysical interactions of saponin on ‘real’ plasma membranes very difficult, simplified artificial membrane systems were designed to mimic both holothuroid and fish plasma membranes. These were put in interaction with the holothuroid saponin Frondoside A.

## Results

### *In vitro* study

#### Thermodynamic analysis of Frondoside-membrane interactions

ITC experiments were conducted on three liposome compositions, fish-like, holothuroid-like and sterol-free lipids, to thermodynamically quantify their interaction with the holothuroid saponin, Frondoside A.

**Figure 1:**
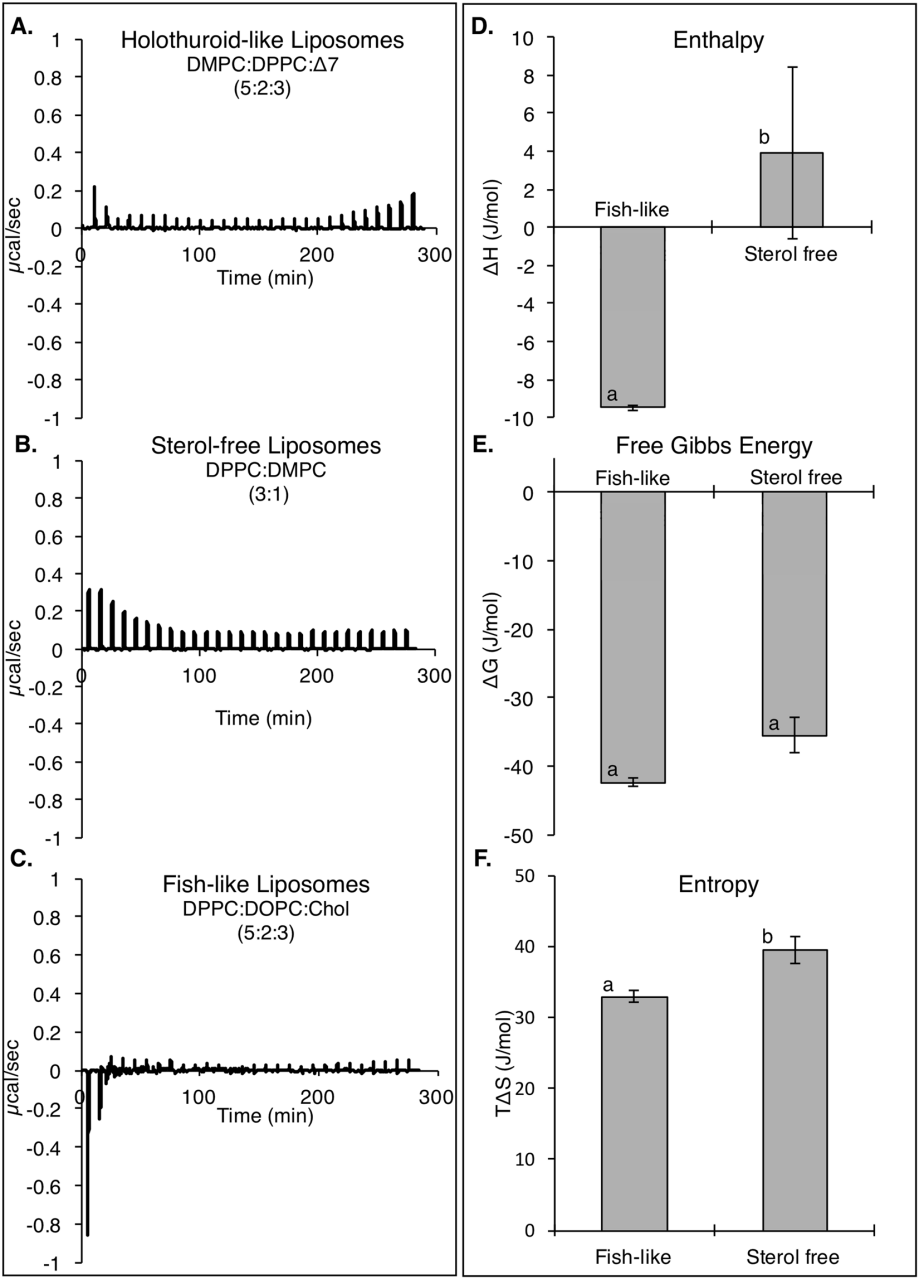
Raw thermograms and calculated thermodynamic characteristics of ITC experiments conducted with the holothuroid saponin Frondoside A and various liposome compositions. (**A**) Holothuroid-like liposomes composed of DMPC:D(C16:1)PC: Δ^7^ sterol (5:2:3) (**B**) Sterol free liposomes composed of DMPC:D(C16:1)PC (3:1) (**C**) and fish-like liposomes D(C16:1)PC:DOPC:Chol (5:2:3). Graphic representation of the thermodynamic values (**D**) Enthalpy (**E**) Entropy (**F**) Free Gibbs Energy, associated with the different ITC experiments. Error bars are standard deviation of replicates (n = 3). Lowercase letters depict significant differences calculated through a pairwise comparison using t-test.

The experiments conducted with fish like liposomes; D(C16:1)PC:DOPC:Chol (5:2:3) resulted in a relatively intense initial negative peak, that decreased and stabilized rapidly over time (Fig. 1C). A different profile was observed for holothuroid-like liposomes; DMPC:D(C16:1)PC:Δ7 sterol, with a weak positive peaks that decreased over time (Fig. 1A), and then an unexpected increase towards the end of the titration. This increase rendered the thermograms impossible to treat analytically, as it is impossible to determine when the titration is complete.

Finally, experiments conducted with sterol-free liposomes; D(C16:1)PC:DMPC (3:1), resulted in thermograms similar to that observed with the holothuroid liposomes, with weak positive peaks decreasing overtime (Fig. 1B).

For the sterol-free and fish like liposomes, the thermodynamic characteristics of the occurring interactions were calculated (Fig. 1D–F). A binding constant of K = 469.6 ± 123.0 mM^−1^ was obtained for fish-like liposomes and of K = 37.4 ± 33.6 mM^−1^ with sterol free liposomes.

The free Gibbs energy of the different experiments were negative for the tested modalities (Fig. 1E). The enthalpy values for the different experiments varied, and were negative for interactions with fish-like liposomes and positive for interactions with sterol-free liposomes (Fig. 1D). Finally, the entropy absolute values were greater than the absolute values of the respective enthalpies (Fig. 1F).

#### Liposome permeability

**Figure 2:**
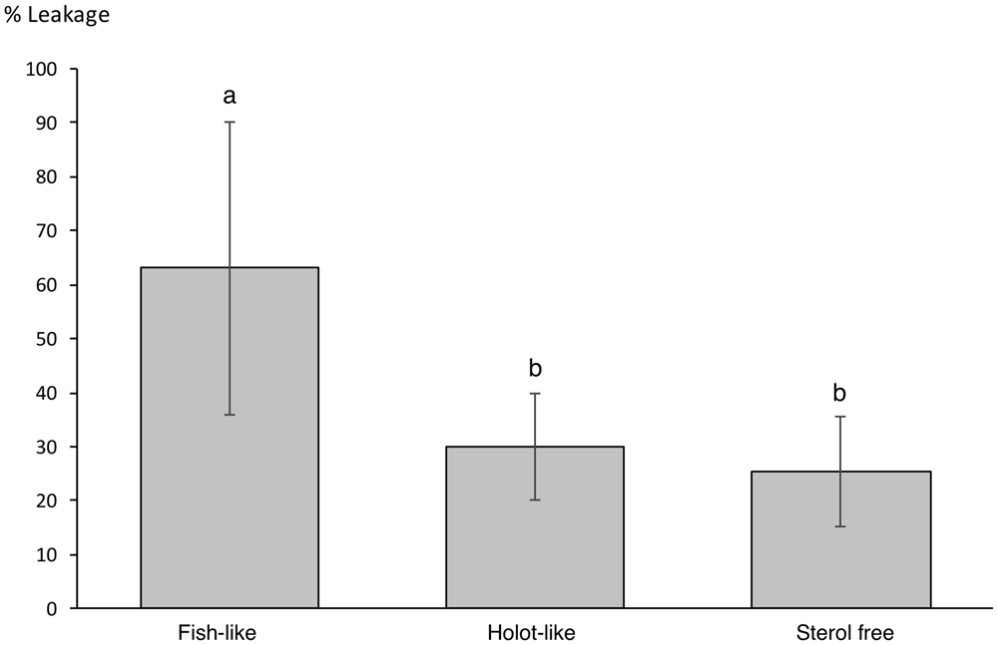
Mean relative leakage of calcein from liposomes of different composition in the presence of the holothuroid saponin Frondoside A. Saponin concentration was 0.4 mM. Fish-like liposomes are composed of D(C16:1)PC:DOPC:Chol (5:2:3); holothuroid-like liposomes are composed of DMPC:D(C16:1)PC: Δ^7^ sterol (5:2:3) and sterol free liposomes are composed of DMPC:D(C16:1)PC (3:1). Error bars are standard deviation of replicates. Lowercase letters depict significant differences calculated through a pairwise comparison using t-test.

To assess the leakage properties of Frondoside A in relation with the lipid composition, permeability assays were carried out. All experiments lead to some level of calcein leakage (Fig. 2). Significant differences were observed between the leakage of fish-like vesicles compared to holothuroid-like (Pairwise comparison using t-test, p = 0.053) and sterol free liposomes (Pairwise comparison using t-test, p = 0.043), in the presence of the Frondoside A solution. However, the level of permeability between holothuroid-like and sterol free liposomes was similar (Pairwise comparison using t-test, p = 0.714).

### *In silico* study

To gain molecular insights for the differences we observed experimentally during liposome-saponin interactions, we used simplified *in silico* modeling approaches to simulate the interaction between Frondoside A and the different sterols and lipids. Due to the lack of experimental data on the 3D structure and properties of this saponin, molecular dynamics calculations were not applicable.

#### Molecular modeling of 3D structure

**Figure 3:**
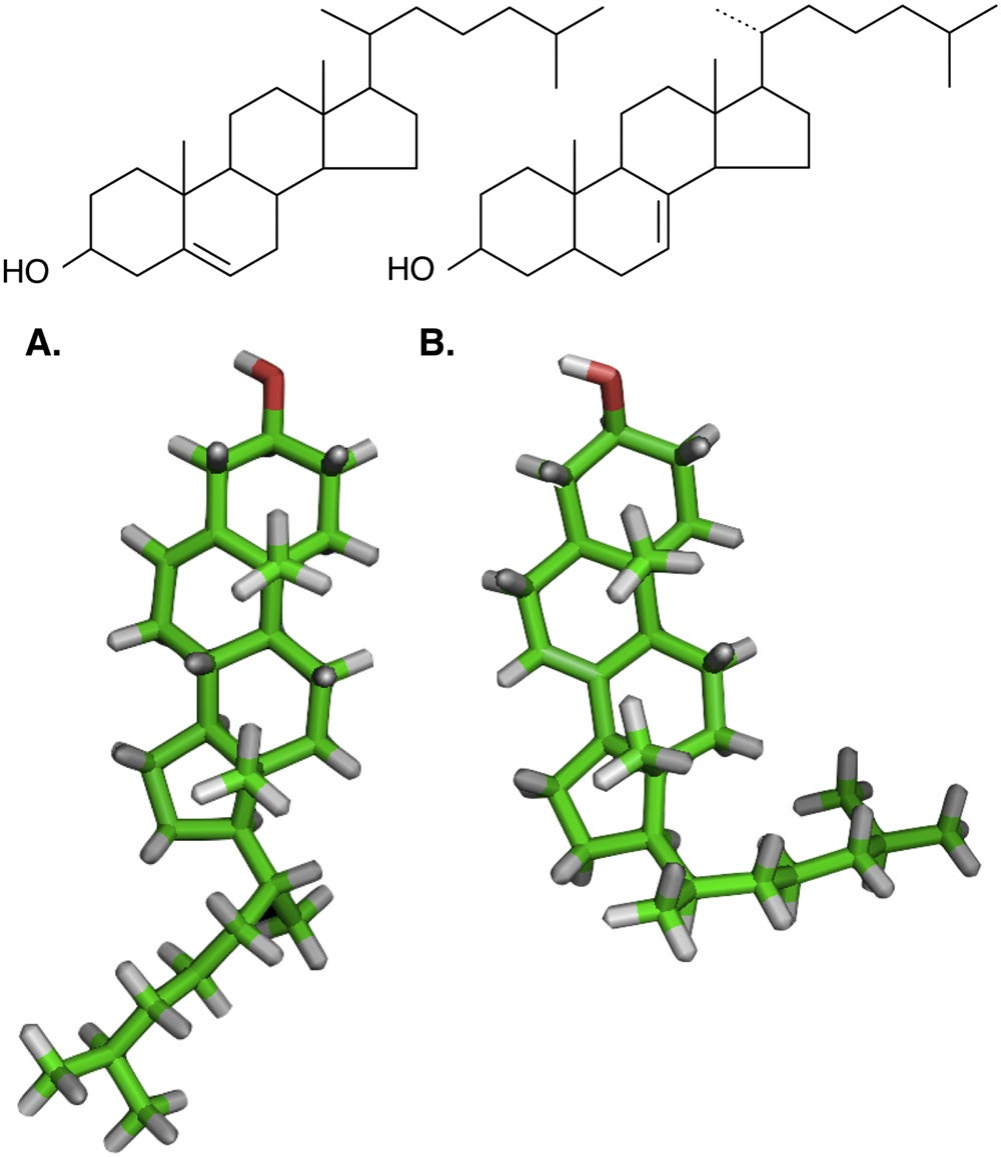
Structural formulae and 3D models of the sterols used in the present study. (**A**) Cholesterol (**B**) 5α-Cholest-7-en-3β-ol (Δ7-sterol). Green: carbon atoms; grey: hydrogen; red: oxygen. The 3D conformations of the two sterols were very different. The Δ7 double bond caused a bend in the molecule generating a “L” shaped sterol. The Δ7 sterol is therefore shorter and wider compared to the Δ5 cholesterol that is relatively elongated. When the interfacial area is calculated, this structural difference is clearly highlighted (51 Å for cholesterol and 78 Å for Δ7).

**Figure 4:**
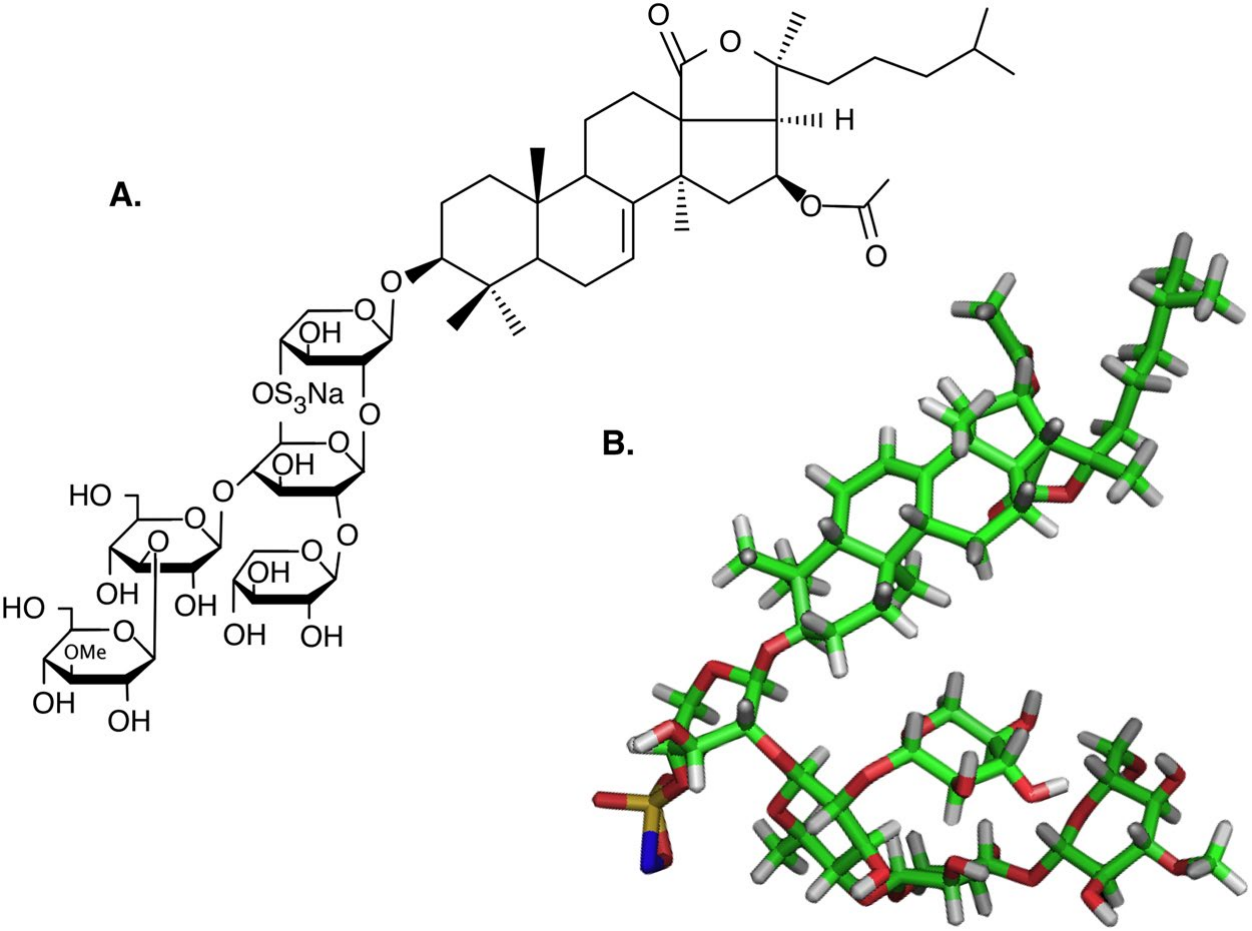
Structure of frondoside A. (**A**) Structural formula; (**B**) 3D model of the holothuroid saponin Frondoside A. Green: carbon; grey: hydrogen; red: oxygen, blue: sodium; yellow: sulfur. Frondoside A had an overall conical shape, with the saccharadic moiety as the base, and the aliphatic chain on the triterpene moiety as the point. The ester bonds of the xylose monomer accommodating the sulphate group were the points of inflection between the two moieties.

The structure of the two sterols under investigation (Fig. 3) and Frondoside A were calculated as described in the methods; their stable 3D structural conformations are depicted in Fig. 4. The calculated 3D structure of the saponin revealed a segregation between the hydrophobic aglycone part of the molecule and the more polar sugar moiety, with each moiety facing each other (Fig. 4).

#### Modeling membrane penetration of Frondoside A

**Figure 5:**
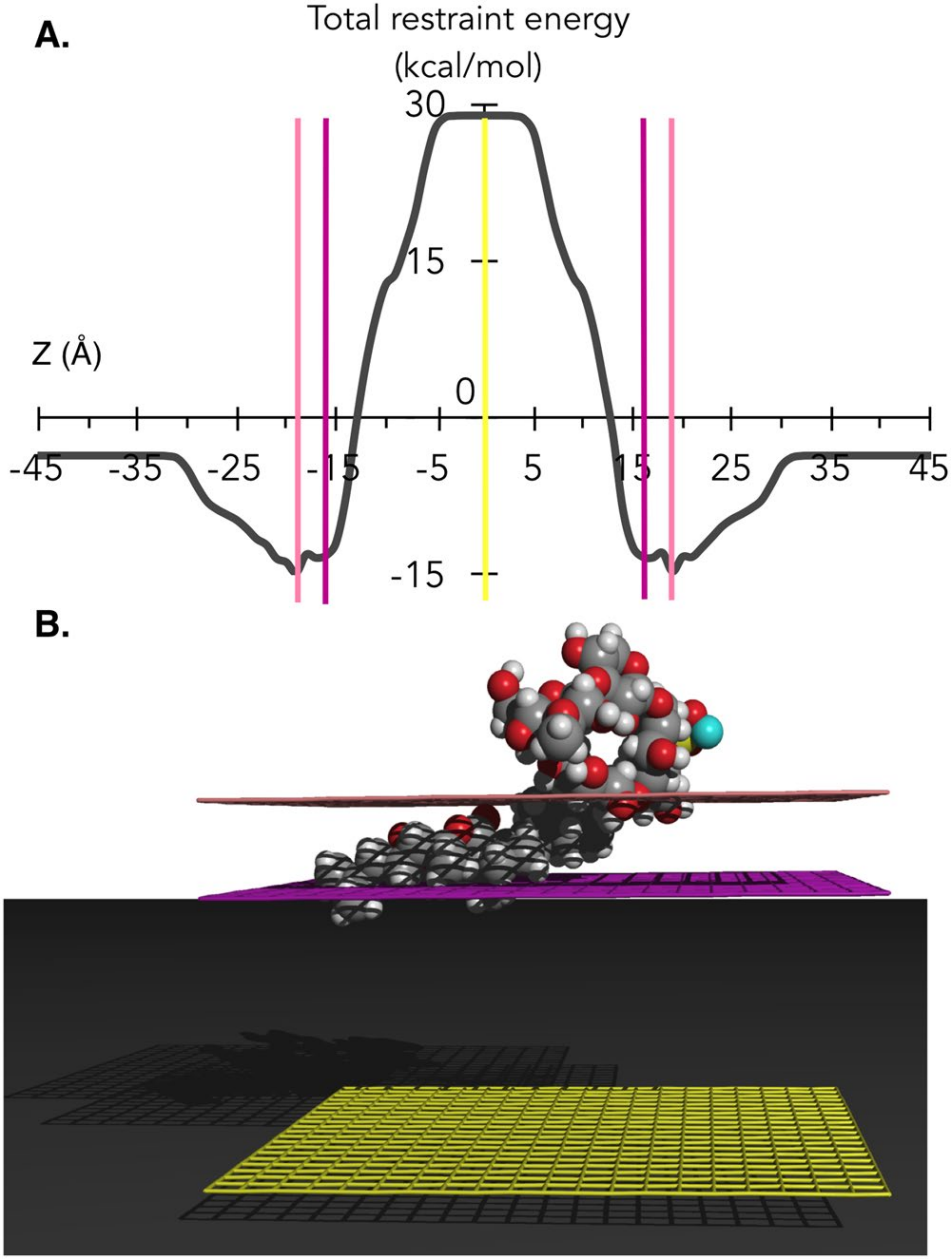
Results from IMPALA simulation of Frondoside A traversing an implicit membrane 36Å thick. (**A**) The “energy-like” profile of the saponin traversing the implicit bilayer. The X-axis corresponds to the position of the center of mass along the Z-axis. The vertical lines represent the same planar surfaces depicted in B. (**B**) The most stable position of the saponin into the implicit bilayer. The different planar surfaces represent the water/membrane interface (pink), the lipid polar head/alkyl chain interface (purple), and the center of the bilayer (yellow). Carbons atoms are dark grey, Hydrogen light grey, Oxygen atoms are red, Sulfur yellow and Sodium light blue.

The propensity of insertion of the saponin molecule into an implicit bilayer was first analyzed. We observed that Frondoside A has its apolar aglycone imbedded in the bilayer (Fig. 5B), its center of mass located near the interface (between 19 and 16 Å for the most stable configurations) (Fig. 5A). Because of the symmetry of the simulated implicit bilayer, the energy profiles were symmetrical.

As would be expected for amphiphilic molecules, positive interaction energies were observed at the hydrophobic center of the bilayer, suggesting that the saponin would not be able to cross the bilayer.

#### Modeling Frondoside-lipid interactions

**Figure 6:**
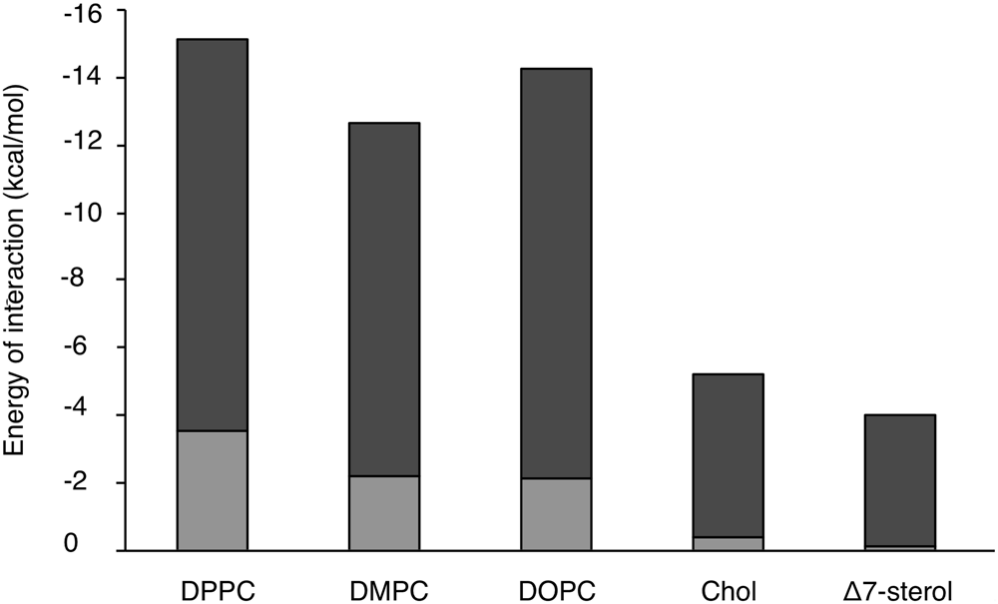
Energy of interaction of Frondoside A and various membrane lipids calculated as described in methods section. Dark grey represents the hydrophobic component and the light grey the polar component of the energies. DPPC: 1,2-dipalmitoyl-sn-glycero-3-phosphocholine, DMPC: 1,2-Dimyristoyl-sn-glycero-3-phosphorylcholine, DOPC: 1,2-Dioleoyl-sn-glycero-3-phosphocholine, Chol: Cholesterol, Δ7-sterol: 5α-Cholest-7-en-3β-ol.

The interaction of Frondoside A with different membrane lipids was investigated using a docking method. In these molecular assemblies, the energies of interaction between the central molecule and the surrounding lipids were calculated (Fig. 6).

The energy values for the interaction between the saponin and phospholipids were more negative than those with sterols. Amongst the sterols, interactions with cholesterol were more favorable than those with Δ7 sterol. For all the molecules tested, the interaction of Frondoside A with lipids are mainly apolar in nature and therefore hydrophobic.

Intra-lipid interaction energies were almost all more favorable in the presence of a saponin (data not shown). In other words, the presence of a saponin in the lipid systems made the systems more energetically stable.

#### Modeling Frondoside-lipid monolayer interactions

We then investigated the behavior and interaction of sterols and phospholipids in the presence of frondoside A in larger more complex monolayer systems. Different lipid monolayers composed of a grid of 200 × 200 molecules were simulated: DMPC/chol/frondo A and DMPC/Δ7 sterol/frondo A at a molar ratio of 63/27/10 and were compared to the plant saponins systems published earlier^29^.

**Figure 7:**
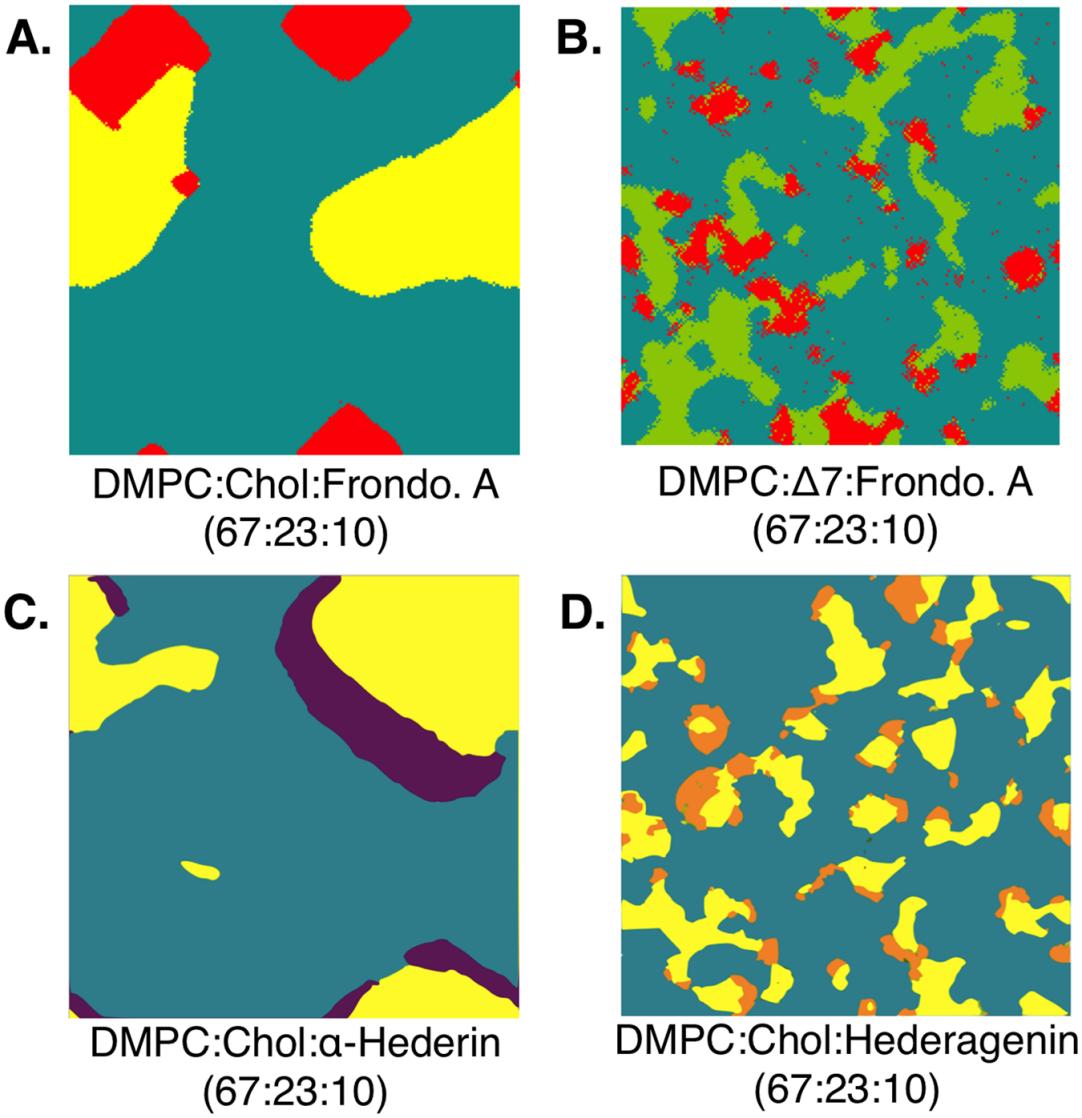
Monolayer simulations of lipid-saponin systems. Each panel is a 200 × 200 grid where each pixel is a molecule. These are obtained by a Monte Carlo minimization for different molecular systems of DMPC, Cholesterol, Δ^7^-sterol, Frondoside A, α-Hederin and Hederagenin. The molar ratios have been selected to be the same as systems published in^28^. Cholesterol is represented in yellow, DMPC in teal, Frondoside A in red, Δ^7^-sterol in green, α-Hederin in purple, and Hederagenin in orange. Panel C and D are adapted adapted with permission from Supporting information of Lorent, J.; Lins, L; Domenech, O.; Quetin-Leclercq, J., Brasseur R. and and Mingeot-Leclercq, M.P. Domain Formation and Permeabilization Induced by the Saponin α-Hederin and Its Aglycone Hederagenin in a Cholesterol-Containing Bilayer. *Langmuir* **30**, 4556–4569 (2014). Copyright (2018) American Chemical Society.

The addition of the saponin Frondoside A to the DMPC:Chol system (Fig. 7A) resulted in very similar changes in cholesterol domain form and size as seen for the plant saponin α-Hederin^29^ (Fig. 7C).

In contrast, the addition of Frondoside A to the Δ7 system resulted into scattered irregularly shaped clusters of Δ7-sterol often associated with the saponin (Fig. 7B). This fragmented sterol profile is similar to that observed for Hederagenin, a sugar-less derivative of the plant saponin α-Hederin (Fig. 7D).

There was a significant difference in relative mean size of the sterol domains between the two systems in the presence of Frondoside A (Fig. 7A,B) (Welch two sample test, df = 4.9905, p-value = 2.885e-06).

## Discussion

### The role of phospholipids in saponin-membrane binding

The cytotoxic nature of Frondoside A is well documented^15,30^, and its potency against cancer cells has caused a rise in interest in this saponin^22,31,32^ and in the membranolytic action of saponins in general.

The amphiphilic nature of saponins, including Frondoside A, allow these molecules to interact with plasma membranes. This was indeed calculated and observed during the *in silico* and *in vitro* experiments conducted in the present study.

ITC experiments with sterol free liposomes resulted in thermograms with positive peaks, distinct to the blank experiments, indicating that Frondoside A was indeed interacting with the liposomes. Similarly, the permeability of sterol free liposomes was greater than the negative controls, further exemplifying interaction between the saponin and sterol free liposomes. In addition, docking calculations suggest a favorable interaction between the saponin and phospholipid molecules. These observations are in agreement with the initial effect of the plant saponin α-hederin on the surface potential of liposomes containing cholesterol or not^33^.

Although some studies have emphasized the absolute necessity of cholesterol in saponin-membrane interactions^10,26,34^, often describing the interaction as the spontaneous formation of cholesterol-saponin complexes, another opinion has emerged and is gaining support. It was first suggested by Brain *et al*.^35^ and then confirmed by others^10,28,29,33^, that saponin-membrane binding can occur independently to the presence of cholesterol, and that phospholipids play an important role in the initial binding phase of these interactions. The thermodynamic interaction characteristics, the permeability induced on sterol-free liposomes and docking simulations presented in the present study are in agreement with this assessment.

### The role of cholesterol in saponin induced membrane permeabilization

Although it would seem that saponin-membrane binding can occur independently to the presence of cholesterol, *in vitro* experiments and the monolayer simulations confirmed previous findings that suggest that the presence of cholesterol is however essential for the strong membranolytic activity of saponins. Permeability assays revealed that cholesterol-containing liposomes were significantly more permeabilized than sterol free liposomes. A similar trend was also observed in the ITC results where there was a relatively intense and rapid exothermic (ΔH < 0) reaction between Frondoside A and cholesterol containing liposomes (fish-like) characterizing the formation of energetically favorable non-covalent interactions between atoms^36^. These interactions are mostly hydrophobic in nature since the absolute value of the entropy component is larger than the absolute value of the enthalpy component, indicating the interactions are entropy driven. The interaction was also characterized by a negative free Gibbs energy, indicative of a spontaneous reaction, and a very large binding constant (400 mM^−1^).

To gain insight into the molecular mechanisms of Frondoside A/cholesterol interaction, we performed simulations with a DMPC/cholesterol monolayer in the presence of Frondoside A, as previously done for α hederin^28,29^. These two saponins induce the same effect, resulting in the progressive sequestration of cholesterol into larger clusters. For α hederin, it was shown experimentally that this ability to induce domain aggregation is correlated to the generation of curvature stress which in turn causes membrane permeabilization and pore formation^33^.

To summarize, the weak but existing interaction of Frondoside A with sterol free liposomes, and the strong destructive interaction of the saponin with cholesterol containing liposomes support the current hypothesis that the cytotoxic nature of saponins is due to the sequence of events: (i) cholesterol-independent binding to the membrane, this step occurs spontaneously, driven by the lipophilic character of the aglycone; (ii) saponins then assemble into complexes with membrane cholesterol (iii) saponin-sterol complexes accumulate into clusters (iv) as a consequence of such accumulation, the steric properties of saponin induce curvature stress, resulting in membrane permeabilization and pore formation, as well as budding and the formation of a new lipid phase containing cholesterol, saponin and phospholipids^10,28,29^.

The molecular mechanism involved in this cytotoxic activity are critical for the potential exploitation of saponins in future therapeutic applications^33^. They are also critical for understanding how holothuroids use saponins as a chemical means of defense against predators, pathogens and competitors. In our study, the same experiments and simulations were extended to holothuroid sterols which allowed us to investigate the roles of these sterols in holothuroid cells that tolerate the cytotoxicity of saponins.

### How sterol structure contributes to saponin tolerance

Although cholesterol and the holothuroid Δ^7^-sterol have the same chemical formula and molecular weight, a 3D rendering of the molecules indicated that the presence of the double bond in Δ^7^-sterol has drastic effects on the 3D conformation. The Δ^7^ bond of 5α-Cholest-7-en-3β-ol resulted in the uplift of the aliphatic chain of the sterols to an almost 90° angle. This is highlighted by the difference in the calculated interfacial surface for each molecule.

The docking calculations further suggested that interactions of Frondoside A were more energetically favorable with cholesterol than with the Δ^7^-sterol.

The different interaction behavior of these sterols towards saponin was also evident in the raw thermograms obtained from the ITC experiments. The interactions with fish- like liposomes were exothermic whereas the interactions of the same saponin with holothuroid-like liposomes were endothermic. If the exothermic interaction is likely linked to increased apolar interactions between the saponin, cholesterol and the PL, the endothermic interactions of saponin with holothuroid-like liposomes suggest the disruption of energetically favorable non-covalent interactions. This could be due to either a local disruption or a gobal rearrangement of the lipid bilayer. This could correspond to the fragmentation of the sterol domains, as seen in the simulated monolayer results.

The monolayer simulations regarding the influence of sterol type on saponin-lipid interaction were most interesting. When Frondoside A is present in the systems, the resulting sterol distribution and cluster size was significantly altered with on the one hand the formation of larger cholesterol clusters, an effect similar to that observed for the plant saponin α-Hederin^29^, and on the other hand the formation of numerous small Δ^7^-sterols clusters. This fragmented sterol profile observed in monolayers composed of DMPC, Δ^7^-sterol and saponin was very similar to that observed for a system of DMPC, cholesterol and a sugar-less α-Hederin derivative, called Hederagenin. Experimental results have shown that Hederagenin had little to no membranolytic activity against model plasma membranes^29^.

It is already known and demonstrated that Frondoside A is cytotoxic^15,30^, and has similar membranolytic activity as the plant saponin α-Hederin^32^. Frondoside A could therefore not be the direct cause for the very different outcome observed between cholesterol-containing and ∆7-containing models. The Δ^7^- sterol hence is likely responsible for the change in membrane activity of Frondoside A observed experimentally. It can therefore be suggested from the current study that Δ^7^-sterols mitigate the “sterol-clustering” activity of Frondoside A, due to its “L-shape”.

It should be noted 4α,14α-dimethyl-5α-cholest-9(11)-en-3β-ol a ∆9(11) sterol, also produced by holoturoids behaves similarly to ∆7 sterol, and has a similar L-shape and has a lower calculated interaction energy with frondoside A, as for ∆7 sterol (data not shown).

## Conclusion

The results obtained in this study strongly suggest that the replacement of cholesterol by biosynthetic precursors such as Δ7 sterol in the cell membranes of sea cucumbers allows these organisms to tolerate the presence of their own cytotoxic saponins. We showed that this tolerance is notably due to the 3D “L” shaped conformation of these sterols, inducing a differential interaction with membrane lipids and a contrasting behavior in the presence of saponins. These differences were consistently brought to light in a series of complementary *in silico* and *in vitro* biophysical experiments. A molecular dynamics approach however may provide deeper understanding into the initial “first encounter” of a saponin with lipid membranes, and the role of phospholipids in particular.

## Materials and Methods

### Chemicals

Various phospholipids and sterols were tested and a fish like, holothuroid like and a sterol free composition were used to produce liposomes for *in vitro* experiments (Table 1).

**Table 1:** Composition of the different liposomes tested for *in vitro* biophysical experiments.

	Sterol free	Holothuroid- like	Fish- like
Lipids	DMPC:D(C_16:1_)PC	DMPC:D(C_16:1_)PC:Δ^7^	D(C_16:1_)PC:DOPC:Chol
Composition	75:25	50:20:30	50:20:30

D(C_16:1_)PC: 1,2-dipalmitoleoyl-*sn*-glycero-3-phosphocholine; DMPC: 1,2-Dimyristoyl-sn-glycero-3-phosphorylcholine; DOPC: 1,2-Dioleoyl-sn-glycero-3-phosphocholine; Δ^7^: 5α-Cholest-7-en-3β-ol.

The holothuroid saponin Frondoside A (Fig. 2) is a triterpene saponin found in the body wall of the sea cucumber *Cucumaria frondosa*^30^. Frondoside A and the holothuroid sterol 5α-Cholest-7-en-3β-ol (Fig. 1) were purchased from Sigma Aldrich (St Louis, MO, USA). The phospholipids and cholesterol were purchased from Avanti Polar Lipids (Alabaster, AL, USA).

### *In vitro* techniques

#### Isothermal Titration Calorimetry (ITC)

Isothermal titration calorimetry (ITC) measures the heat released (exothermic reaction) or absorbed (endothermic reaction) when two interacting components are brought together using a VP-ITC (MicroCal, Northampton, MA). This technique provides a complete thermodynamic description of binding processes^37–39^. In the present study, a solution of the holothuroid saponin Frondoside A (Sigma-Aldrich®), was in the measuring cell of the ITC, and a solution of liposomes was in the titrating syringe^40,41^.

In addition, two types of blanks were conducted and subtracted from the raw thermograms to reveal the actual interaction thermogram. It was observed that the blank experiment conducted by titrating the liposomes solution into the buffer resulted in systematic negative peaks whereas the blank experiment conducted by titrating buffer into a solution of Frondoside A resulted in systematic positive peaks.

Large unilamellar vesicles (LUVs) of various compositions mimicking different types of cell plasma membranes (Table 1) were prepared by extrusion as described by Zakanda *et al*.^40^. During the preparation of the liposomes a saline-Tris-HCl buffer (600 mM NaCl, 10 mM TRIS, pH 7.5) was used to mimic marine conditions. The size and homogeneity of the produced liposomes was verified by Dynamic Light Scattering. Sterol free liposomes had an average diameter of 131.3 ± 4.9 nm, holothuroid-like liposomes were of a diameter 176.63 ± 4.61 nm and fish- like liposomes had a diameter of 151.1 ± 3.1 nm.

Titration was carried out at 26 °C using a 300 μL syringe filled with the LUV suspension at 5 mM. The saponin solution (15–25 uM) in the sample cell was stirred at 305 rpm during the experiments. A titration experiment consisted of consecutive injections of 10 μL of the LUV suspension. Each injection took 10 s and a delay of 360 s was applied between each successive injection to allow steady state to be attained. Data were processed using the software provided by the manufacturer (ORIGIN 7, Originlab, Northampton, USA).

#### Calcein Release from LUVs

The leakage of entrapped self-quenched calcein from LUVs induced by a permeabilizing agent can be monitored by the increase of fluorescence caused by its dilution^33,40^. LUVs of fish-like, holothuroid-like and sterol-free (Table 1) were prepared in a saline-Tris-HCl buffer (600 mM NaCl, 10 mM TRIS, pH 7.5) with a self-quenching concentration of calcein (10 mM). The un-encapsulated dye was removed on a gel column of Sephadex G75^42^.

The purified calcein-filled liposomes were put in contact with either a 0.5% solution of Triton-X as a “maximal” permeabilizing agent, with methanol as a “minimal” permeabilizing, and with a 0.4 mM methanol solution of Frondoside A. The excitation and emission wavelengths were 472 and 512 nm, respectively, and the fluorescence was measured over a period of 900 sec.

The percentage of released calcein was calculated using the formula:

1 $$\begin{array}{c} \% Leakage=[\frac{({F}_{t}-{F}_{contr})}{({F}_{tot}-{F}_{contr})}]\end{array}\times 100$$

where, F_t_ is the fluorescence signal for a given concentration of saponin. F_contr_ is the fluorescence signal for liposomes with methanol. F_tot_ is the fluorescence signal of liposomes incubated with 0.5% Triton X-100.

The saponin solution concentrations used during the *in vitro* techniques were optimized so that measurements would fall between the lower limit of measurement and the limit of saturation.

### *In silico* techniques

#### Structure tree method

The conformation of Frondoside A and the sterols of interest were determined by considering the main torsional angles of the molecules and intra-molecular energies of interaction, using an empirical force field^43^. The most probable conformation was obtained as previously described^44^. This force field takes into account the hydrophobic energy and therefore allows to obtain 3D structure for small molecules in which hydrophobic interactions are optimized, as assessed in^44^.

#### Hypermatrix method

The Hypermatrix method^45,46^ is a simple docking method that allows for the calculation of the interaction between a molecule and lipids. The molecule of interest (saponin in this study) is fixed at the center of the system and oriented at the hydrophobic (pho)/hydrophilic (phi) interface using the TAMMO procedure^46^. The lipid molecules are also oriented at the pho/phi interface and, by a succession of rotations and translations of the lipids around the central molecule, the energy of interaction of over 10^7^ positions are calculated using the force field described by Lins and Brasseur (1995)^44^. Lipids are placed around the central molecule one at a time taking into account the steric and energetic constraints caused by the presence of the previously placed lipids. To avoid bias in the calculated energies of interactions due to the size of the lipids, the interaction energy and its composing terms (EVdW, Electrostatic, hydrophobic) were divided by the number of heavy atoms of each molecule considered. This docking method provides information on the affinity of the central molecule for the surrounding lipids, and can be compared between systems. The method also allows to calculate the interfacial area of each molecule by a projection on the interfacial plane^37^.

#### IMPALA method

The IMPALA method as first described by Ducarme *et al*.^47^ uses a membrane model in which the membrane bilayer is implicitly modeled by an empirical function C(z). This model assumes that the properties of the implicit membrane are constant in the X,Y plane and only vary along the perpendicular Z (in Å) axis that originates at the bilayer center. C(z) varies from 1 (completely hydrophilic) to 0 (completely hydrophobic)^47^.

2 $$\begin{array}{c}C(z)=1-\frac{1}{1+{e}^{\alpha (|z|-{z}_{0})}}\end{array}$$

where α is a constant equal to 1.99, z_0_ is the position of the hydrophilic/hydrophobic interface in the membrane and z is the position in the membrane. The total thickness of the bilayer was set at 36 Å^47^.

The IMPALA method uses two energy restraints^46^, a hydrophobic restraint and a lipid perturbation restraint, to simulate the interactions between the molecule of interest and the lipid bilayer. The saponin is translated across the implicit bilayer along the Z axis 1 Å at a time and rotated 360° at each position z(i), and the sum of the two restraints is calculated to predict the most stable position into the implicit membrane.

#### Modeling saponin-lipid monolayer interactions- Big Monolayer method

This approach is derived from the Hypermatrix method but increases the number of interacting partners and therefore the total number of molecules in the system^48^. The method is based on the construction of a grid of 200 × 200 molecules and its minimization by a Monte Carlo procedure^29,48^ using the interaction matrix calculated for each pair of molecules (saponin/saponin, saponin/sterols, sterols/DMPC, DMPC/DMPC) as described above. Graphically, each molecule type is represented by a colored point and all the points were drawn on the grid. This allows to visualize preferential interactions and phase separation between molecules and was successfully applied for plant saponins^29^.

For the purpose of this study, the monolayer compositions were derived from Lorent *et al*.^29^ *i.e*. 67%DMPC, 23% sterol, 10% saponin, in order to compare results. Each calculation, with and without saponin, was repeated 5 times.

Image analysis of the resulting colored grids was conducted using the software Fiji^49^. The particle analysis tool was used to determine the surface area of the different domains/clusters. The average sterol domain size was first divided by the total area occupied by sterol, to relativize the data between systems of different composition. In addition, because of large differences in variances between compared experiments, the distribution of the mean was normalized using the log of values. A Welch’s two sample test was used to compare the mean particle areas of the sterol clusters, using the software R^50^.
